# Mind-Body Approaches to Prevention and Intervention for Alcohol and Other Drug Use/Abuse in Young Adults

**DOI:** 10.3390/medicines5030064

**Published:** 2018-06-27

**Authors:** Crystal L. Park, Beth S. Russell, Michael Fendrich

**Affiliations:** 1 Department of Psychological Sciences, University of Connecticut, Storrs, CT 06269, USA; 2 Human Development & Family Studies, University of Connecticut, Storrs, CT 06269, USA; Beth.russell@uconn.edu; 3 School of Social Work, University of Connecticut Hartford, Connecticut, CT 06103, USA; Michael.fendrich@uconn.edu

**Keywords:** substance use, alcohol misuse, emotion regulation, mindfulness, meditation, yoga

## Abstract

Alcohol and other drug (AOD) misuse is highly prevalent among young adults and creates myriad long-term problematic social, economic, and health consequences. Current treatments aimed at preventing or alleviating AOD misuse have demonstrated fairly inconsistent and weak effectiveness and, thus, are far from a complete solution. In this review, we describe the current state of AOD interventions for young adults and present an alternative emotion regulation framework for understanding AOD use/misuse. We then describe implications of this framework for interventions to promote healthier emotion regulation to successfully reduce AOD use/misuse. In particular, we assert that mind–body approaches, such as meditation, distress tolerance, and yoga, may promote emotion regulation skills that allow young adults to manage their stressful experiences and distressing emotions without AOD use. We review the available literature on mind–body interventions targeting AOD use/misuse in young adults and offer suggestions for future intervention development and research.

## 1. Problematic Alcohol and Other Substance Use in Young Adults

Both alcohol and other drug (AOD) use are highly prevalent in the US and are associated with many adverse consequences, many of which manifest in young adulthood. Excessive alcohol use is the fourth leading cause of death in the United States [1]. Results from the most recent National Survey on Drug Use and Health (NSDUH) [2] indicate that 18–25-year-old respondents reported the highest alcohol use rates (over 58%) as well as the highest rates of heavy episodic or “binge” drinking of any age group. Over one-third of all 18–25-year-old participants indicated that they had consumed 4–5 drinks or more in a single occasion during the previous 30 days. The prevalence rate of heavy episodic drinking for this age group is at 34%, which is just below the highest historical rates ever recorded by this survey. Problematic alcohol use in young adults is associated with myriad negative consequences, including fatal and nonfatal injuries [3], physical violence against others [4], sexual assault victimization and perpetration (e.g., [5,6,7]), being bullied [8], increased risky sexual behavior (e.g., [9,10]), mental health problems (especially elevated depression [11,12]), and decreased odds of post-graduation employment—post-graduate employment is typically a goal of undergraduate college students [13].

Drug-related deaths continue to be on the rise in the US [14] with age-adjusted overdose death rates increasing 15.6% from 2014 to 2015 and 21.5% from 2015 to 2016 [14]. These increases were observed across all age groups and across multiple substances but were particularly evident among young adults in their mid-twenties [14]. Results from the 2015 *Monitoring the Future* national survey on drug use [15] suggest that “by their late 20s, nearly two-thirds (63%) of today’s young adults have tried an illicit drug, and about four in ten (37%) have tried some illicit drug other than marijuana, usually in addition to marijuana” [15] (p. 38). College students have the highest prevalence of any age group of illicit drug use (41%)—higher than their non-collegiate 19–28-year-old peers or high school-aged adolescents. Their prevalence rate for illicit drug use other than marijuana use is also the highest, tied at ~20% for both college students and their 19–28-year-old non-collegiate peers. Rates of marijuana use from 2014 to 2015 increased to 34% among the general young adult population and to 28% among college students—the highest rates recorded in 25 years.

The National Comorbidity Survey (NCS) underscores that AOD use initiated in the period of young adulthood is not just experimental and recreational but rather may have lasting effects on consumption trajectories: For the majority of adults diagnosed with a substance use disorder, onset occurred during young adulthood [16]. Effective interventions for young adults are crucial in preventing the escalation of AOD misuse into disordered behavior, and there is growing consensus that not all intervention approaches are equally effective across the heterogeneous population of substance-using young adults. The development of biopsychosocial models customized to individual patient’s needs and characteristics are needed for the prevention of AOD misuse. For example, recent advances in substance use research suggest that chronic and severe substance-using populations may differ in important ways (e.g., specific types of cognitive impairments), which is informative in tailoring treatment approaches (e.g., chronic and severe marijuana, alcohol, and cocaine users [17]).

## 2. Current Approaches to Reducing Substance Use in Young Adults

Research suggests that there are possibly at least four different motives that may drive heavy (and problematic) AOD use: coping, enhancement, social, and conformity (peer pressure) [18]. Although using AOD to cope (i.e., coping motives, which relate to the relief of anxiety or depression) has been identified as important, current widely-used AOD misuse intervention programs (for example, Brief Alcohol Screening and Intervention for College Students, BASICS, and Marijuana Abuse Prevention Program, MAPP [19]) are typically based on the idea that students’ AOD Misuse is influenced by the conditions of the college environment where students feel pressure to fit in and conform. These programs often view the college environment as one where AOD misuse is prevalent and perceived as normative. 

Accordingly, these collegiate intervention programs aim to enhance individuals’ self-efficacy and motivation to “act responsibly” with respect to AOD use [20]. Treating problematic substance use as a misinformed choice, these programs employ brief motivational interventions (BMIs) to provide personalized feedback with regard to the consequences of use, normative (mis)perceptions about other students’ use, and expectancies regarding the effects of substance use. Many collegiate substance use prevention programs rely on intervention curricula informed by BMI approaches, some employing web-based adaptations, which have demonstrated modest efficacy with respect to short-term reductions in alcohol use [20], but minimal long-term impact [21]. Evidence regarding the impact of BMIs on drug use, especially among those who are not identified as also having problematic alcohol use, is also mixed [22] with several controlled studies [23,24], suggesting limited or no impact. These inconsistent and short-term effects may be the result of an intervention approach that only partially addresses a subset of the underlying factors leading to young adults’ AOD misuse. In particular, we assert that promoting more effective coping skills to regulate negative emotions might be a useful intervention approach for reducing use and misuse of AOD.

## 3. An Emotion Regulation Perspective on Young Adults’ AOD Use/Abuse

Emotion regulation (ER) refers to “extrinsic and intrinsic processes responsible for monitoring, evaluating, and modifying emotional reactions, especially their intensive and temporal features, to accomplish one’s goals” [25] (p. 27). During young adulthood, critical changes in ER occur, particularly age-related shifts in strategies used to manage distress (e.g., emotion suppression, inhibitory control, and cognitive reappraisal [26,27]). The effortful or planful and strategic management of distress is quite salient in young adulthood, a developmental period marked by an immature emotion regulatory system with heightened reward sensitivity and less inhibition of impulsive behavior [28,29,30,31,32]. Substantial evidence suggests that deficits in ER are strongly related to patterns of AOD use in young adults [33,34,35,36].

In particular, deficits in the self-regulation of discomfort and distress, called *distress tolerance*, predict use—specifically, motivation and urgency for use, escalations in consumption, and the development of dependence that may indicate substance use disorders (SUDs) [33,37]. Copious research has linked substance use specifically to difficulties in ER, describing substance use as a strategy individuals use to escape, avoid, or regulate emotional discomfort [38]. Young adults who lack more adaptive ways to regulate emotions may turn to substances as a way to minimize or numb negative emotions. Substance use prevention and treatment efforts then are best able to address the relationships between substance use and mood by considering how emotional experiences underlie or exacerbate maladaptive coping strategies. As noted by Dingle and colleagues [39], “even following treatment, emotional distress is the number one trigger for relapse into substance misuse” (p. 187). Thus, learning ER skills, particularly the management of distress, holds significant promise in preventing the escalation of AOD misuse to SUDs in young adulthood.

Impaired ER is associated with substance use initiation and on-going use despite negative consequences, setting the stage for substance use disorders [30,40]. Moreover, AOD misuse may be a coping strategy for young adults unable to otherwise regulate discomfort and distressing emotions [41,42]. In turn, substance use may impair the development of executive functions [17]. In sum, impaired and still-developing ER skills in young adulthood comprise a complex constellation of initiating and exacerbating factors occurring in a sensitive developmental period [43,44].

## 4. Mind–Body Interventions for Emotion Regulation in the Context of Substance Use/Abuse

Importantly, social norms and BMI interventions do not address ER difficulties, a primary driver of AOD misuse that can persist beyond the college years and far into adulthood. Given the incomplete and variable effectiveness of these approaches for reducing young adults’ substance misuse [21,45], there is a clear need for alternative intervention approaches. Research on the strong associations between ER and AOD use in young adults [33,34] suggests that attending to their individual resources and coping skills may be critical for effective interventions to prevent escalating substance misuse. Indeed, we know from an extensive literature that chronic stress creates vulnerability to addiction (e.g., [46]) and impacts alcohol use and other related behaviors (e.g., [47]). Accordingly, when young adults learn ER skills—particularly when they learn how to effectively manage discomfort and distress—these skills hold significant promise in stemming the escalating AOD use towards SUDs.

## 5. Mind–Body Approaches to Bolstering ER Skills to Prevent AOD Use/Misuse

Individuals regulate their emotional distress and discomfort associated with stressors in a myriad of adaptive or maladaptive ways, often including AOD use. Emotion regulation depends on an individual’s unique mental representations of everyday and stressful events through which they interpret and navigate daily life [48]. Within this framework, self-evaluation is critically important in order to respond adaptively to distressing emotions. For example, the ability to monitor one’s efforts to regulate or manage discomfort or distress and evaluate the effectiveness of these attempts are undergirded by a mindful ongoing awareness of one’s physical and emotional state. Further, possessing coping skills that allow individuals to reappraise discomfort or distress as manageable, as well as having adequate internal resources such as self-efficacy and control, also allow individuals to more adaptively regulate their emotions [49,50].

Thus, interventions that target ER by bolstering self-reflection/evaluation, imparting ER skills, and increasing internal resources present opportunities to build resilience [50]. Mind–body interventions for AOD misuse, which aim to prevent escalations by acknowledging the influence of physical and mental health within the social context of young adult life, do just this and are a natural adjunctive approach to those focused on social influence to create a more comprehensive strategy to AOD intervention. To date, mind–body approaches have not been extensively applied to AOD use and misuse, and very little of this research has been conducted with young adults. In the section below, we review the literature on mind–body interventions focused on emotion regulation in young adults, with a focus on those targeting AOD use. In particular, we review mindfulness approaches to increasing ER in young adulthood focusing on distress tolerance interventions and yoga-based interventions.

## 6. Distress Tolerance Interventions to Improve ER and Reduce AOD Use/Misuse

Studies have convincingly demonstrated the effectiveness of ER interventions to impact dysregulated behavior, noting these efforts are promising because they focus on individual’s struggles to regulate themselves when experiencing intense negative emotions [51,52,53]. Specifically, distress tolerance (DT) represents a promising ER mechanism for preventing AOD use [34,50,54,55]. DT interventions focus on equipping individuals with ER skills that encourage deescalating an intense or impulsive emotional response to distress to a calmer, more mindful response. This response is more likely to include adaptive coping strategies and less likely to include impulsive or harmful responses to distress, including substance use.

Mindfulness and meditation activities common in these efforts promote emotional clarity by training individuals to attend to present experiences non-judgmentally [56]. With practice, individuals who can mindfully attend to their emotions experience fewer moments of crisis and build a sense of competence in regulating their distress—thereby becoming more self-efficacious in their resilience [57]. Similar to most mindfulness-based interventions, DT interventions are often didactic social small group interventions that present activities and conversation topics to promote ER [58]. Participants in these interventions are taught self-reflection, non-judgmental awareness of their experiences, and adaptive coping strategies—often through meditation and similar reflective practices such as guided or self-visualization—in an effort to promote self-management and health and wellbeing.

Development of mindfulness-based approaches to AOD use in young adults is in a very early stage but results so far are promising, suggesting that increasing mindfulness may reduce negative affect and subsequent urges to engage in AOD use [18,59,60,61,62,63]. For example, a two-session mindfulness and motivational interviewing intervention demonstrated reduced marijuana use over three months in young women [64].

Building on these more general mindfulness approaches to young adult AOD use, DT has demonstrated significant associations with lower levels of substance use [65,66,67,68,69,70,71]. This evidence suggests DT is a significant correlate and moderator of AOD use. However, there is a *noteworthy gap* in the literature specific to young adults. To date, we are aware of only one randomized controlled trial of a DT intervention to reduce substance use in *any* age group: In a sample of 49 individuals (mean age 40 years), Stein and colleagues [72] reported positive effects from their DT training series of seven 50-min sessions. Given the shifts in ER during this stage of development and the high rates of AOD use in young adulthood leading to SUDs, more work in this area is sorely needed.

## 7. Yoga to Improve ER and Reduce AOD Use/Misuse

As noted above, ER involves self-awareness, cognitive reframing, and mindfulness, all of which increase one’s capacity to control, override, or accept spontaneous negative emotional responses [50]. Yoga has been suggested to be an exceptional path towards skillful ER [73] and thus may provide a highly effective route towards alleviating AOD use/misuse. Many of the beneficial effects of yoga on physical, mental, and spiritual well-being can be attributed to its fostering a greater capacity for ER.

Classical yoga was originally designed to create mind–body harmony and aid in the ultimate goal of “enlightenment” [74]. In the past century, yoga has evolved considerably, with much greater emphasis placed on the physical aspects of yoga postures (asanas) and practice. Hatha yoga, the modality most often practiced in the United States, involves the practice of yoga postures together with breathing techniques and concentration/meditation [73]. Many aspects of yoga practice help to develop and support ER [75], including meditative movement, conscious breathing, body and emotion awareness, open curiosity, attention allocation, self-compassion, and acceptance [73,76]. Through these aspects of yoga, practitioners can strengthen their capacity for emotional stability and equanimity, an even-minded mental state or dispositional tendency toward all experience regardless of its affective valence or source. Through continued practice, yoga can help individuals cultivate more adaptive and fewer maladaptive cognitions, emotions, and behaviors. In combination, these different aspects of yoga make it a highly potent intervention for improving self-regulation capacity. With more yoga practice, practitioners develop greater tendencies for adaptive ER, both on and off the yoga mat.

Yoga interventions for ER have demonstrated feasibility in a variety of settings, including a small number of studies conducted with young adults [76,77,78,79]. Based on these and other studies, Khanna and Greeson [80] noted that “the skills, insights, and self-awareness learned through yoga and mindfulness practice can target multiple psychological, neural, physiological, and behavioral processes implicated in addiction and relapse. A small but growing number of well-designed clinical trials and experimental laboratory studies on smoking, alcohol dependence, and illicit substance use support the clinical effectiveness and hypothesized mechanisms of action underlying mindfulness-based interventions for treating addiction” (p. 244). For example, a randomized controlled trial found that alcohol-dependent participants in an eight-week yoga intervention had greater declines in dependence severity compared to participants in the physical training exercise control condition [81]. Among clients in an outpatient methadone program, yoga interventions have been shown to be as effective as traditional group psychotherapy for reducing substance use [82]; similarly, a yoga intervention in a military population was associated with significantly reduced rates of alcohol and substance use [83].

To date, however, no research has examined the utility of a yoga intervention to reduce AOD use/misuse in young adults, perhaps because people who engage in substance misuse are typically excluded from studies of yoga interventions [84]. Results of a recent national survey indicate that nearly 20% of young adults in the US aged 18–29 currently practice yoga [85]; as yoga continues to grow in popularity in the United States, the study of its effects on ER and AOD has the potential to make an important and timely contribution to developing more effective substance use interventions for young adults. 

## 8. Issues for Future Development of Mind–Body Approaches to Reducing AOD Use in Young Adults

Given the broad and burgeoning interest in meditation and yoga interventions for a host of mental and physical disorders [86,87], it is somewhat surprising that so little research has investigated their potential efficacy for treating AOD use/misuse in the high-risk group of young adults. Young adults are quite interested in meditation and yoga. For example, the number of young adults who had practiced yoga in the past year had increased to nearly 30% in the most recent NHIS survey [88]. Given this interest, these approaches may have broad appeal and acceptability. Clearly, this is an area warranting substantial research attention, given the promising findings regarding their efficacy for many other conditions [89] and the direct effects they have demonstrated on ER skills.

Further, research is needed on how these complementary approaches might best be integrated into existing interventions for young adults—including on college campuses where over 70% of young adults pass through [90]—given the wide reach of existing interventions. While the efficacy of existing programs may be limited, they have widespread institutional acceptance in the US and have been easy (and cost-effective) to implement. Indeed, some of these traditional interventions have been adapted so that they can be accessed online through personal electronic devices (e.g., Alcohol e-CHeckUpToGo (e-CHUG) [91]). DT- and yoga-based approaches currently require face-to-face interaction and skilled trainers need to be available to work with live groups of students. However, new technologies are being developed to deliver such interventions remotely, both synchronously and asynchronously [92]. Can young adults be cost-effectively treated with such interventions, given the limited budgets of many institutions of higher education and our health care system? Is it possible to “mass produce” yoga and DT interventions in the same way that existing BMI interventions have been developed nationwide in the US? These implementation issues require further exploration in future research.

Finally, any potential implementation of yoga/DT interventions would require assessment of AOD outcomes as well as of theoretically informed measures reflecting the potential processes that underlie AOD use. Thus, researchers should measure levels of DT and ER as well as other variables that potentially may impact substance use and substance use motives. This assessment would be especially important if the yoga/ER interventions were developed as an adjunct to existing BMI or social norm programming. Enhancement of programming requires a clear understanding of the mechanisms of action and such an understanding requires careful measurement not only of outcomes but of intervening processes. With this comprehensive measurement and the resulting refinement of theory, we can develop improved programming to more effectively address the problem of AOD use/misuse in young adults.

## References

[1] Stahre M., Roeber J., Kanny D., Brewer R.D., Zhang X. (2014). Contribution of excessive alcohol consumption to deaths and years of potential life lost in the United States. Prev. Chronic Dis..

[2] Lipari R., Jean-Francois B. (2016). Trends in Perception of Risk and Availability of Substance Use among Full-Time College Students.

[3] Fendrich M., Berger L., Fuhrmann D. (2017). The association of long-term alcohol biomarkers with risk for alcohol-related injury: Implications for screening. J. Subst. Use.

[4] Wechsler H., Davenport A., Dowdall G., Moeykens B., Castillo S., Frances R.J. (1996). Health and Behavioral Consequences of Binge Drinking in College: A National Survey of Students at 140 Campuses. Yearb. Psychiatry Appl. Ment. Health.

[5] Testa M., Livingston J.A. (2009). Alcohol consumption and women’s vulnerability to sexual victimization: Can reducing women’s drinking prevent rape?. Subst. Use Misuse.

[6] Abbey A. (2002). Alcohol-related sexual assault: A common problem among college students. J. Stud. Alcohol.

[7] Mallett K.A., Lee C.M., Neighbors C., Larimer M.E., Turrisi R. (2006). Do we learn from our mistakes? An examination of the impact of negative alcohol-related consequences on college students’ drinking patterns and perceptions. J. Stud. Alcohol.

[8] Rospenda K.M., Richman J.A., Wolff J.M., Burke L.A. (2013). Bullying victimization among college students: Negative consequences for alcohol use. J. Addict. Dis..

[9] Hutton H.E., McCaul M.E., Santora P.B., Erbelding E.J. (2008). The relationship between recent alcohol use and sexual behaviors: Gender differences among sexually transmitted disease clinic patients. Alcohol. Clin. Exp. Res..

[10] Brown J.L., Vanable P.A. (2007). Alcohol use, partner type, and risky sexual behavior among college students: Findings from an event-level study. Addict. Behav..

[11] Geisner I.M., Mallett K., Kilmer J.R. (2012). An examination of depressive symptoms and drinking patterns in first year college students. Issues Ment. Health Nurs..

[12] Geisner I.M., Mallett K., Varvil-Weld L., Ackerman S., Trager B.M., Turrisi R. (2018). An examination of heavy drinking, depressed mood, drinking related constructs, and consequences among high-risk college students using a person-centered approach. Addict. Behav..

[13] Bamberger P.A., Koopmann J., Wang M., Larimer M., Nahum-Shani I., Geisner I., Bacharach S.B. (2018). Does college alcohol consumption impact employment upon graduation? Findings from a prospective study. J. Appl. Psychol..

[14] Seth P., Scholl L., Rudd R.A., Bacon S. (2018). Overdose deaths involving opioids, cocaine, and psychostimulants—United States, 2015–2016. Morb. Mortal. Wkly. Rep..

[15] Johnston L.D., O’Malley P.M., Bachman J.G., Schulenberg J.E., Miech R.A. (2015). Monitoring the Future National Survey Results on Drug Use, 1975–2014.

[16] Kessler R.C., Demler O., Frank R.G., Olfson M., Pincus H.A., Walters E.E., Wang P., Wells K.B., Zaslavsky A.M. (2005). Prevalence and treatment of mental disorders, 1990 to 2003. N. Engl. J. Med..

[17] Aharonovich E., Campbell A.N.C., Shulman M., Hu M., Kyle T., Winhusen T., Nunes E.V. (2018). Neurocognitive profiling of adult treatment seekers enrolled in a clinical trial of a web-delivered intervention for substance use disorders. J. Addict. Med..

[18] Vernig P.M., Orsillo S.M. (2009). Psychophysiological and self-reported emotional responding in alcohol-dependent college students: The impact of brief acceptance/mindfulness instruction. Cogn. Behav. Ther..

[19] Dimeff L.A. (2009). Brief Alcohol Screening and Intervention for College Students (BASICS): A Harm Reduction Approach.

[20] Cronce J.M., Bittinger J.N., Liu J., Kilmer J.R. (2014). Electronic feedback in college student drinking prevention and intervention. Alcohol Res. Curr. Rev..

[21] Carey K.B., Scott-Sheldon L.A., Elliott J.C., Carey L., Carey M.P. (2012). Face-to-face versus computer-delivered alcohol interventions for college drinkers: A meta-analytic review, 1998 to 2010. Clin. Psychol. Rev..

[22] Dennhardt A.A., Murphy J.G. (2013). Prevention and treatment of college student drug use: A review of the literature. Addict. Behav..

[23] Elliott J.C., Carey K.B. (2012). Correcting exaggerated marijuana use norms among college abstainers: A preliminary test of a preventive intervention. J. Stud. Alcohol Drugs.

[24] Lee C.M., Neighbors C., Kilmer J.R., Larimer M.E. (2010). A brief, web-based personalized feedback selective intervention for college student marijuana use: A randomized clinical trial. Psychol. Addict. Behav..

[25] Thompson R.A. (1994). Emotion regulation: A theme in search of definition. Monogr. Soc. Res. Child Dev..

[26] McRae K., Gross J.J., Weber J., Robertson E.R., Sokol-Hessner P., Ray R.D., Gabrieli J.D.E., Ochsner K.N. (2012). The development of emotion regulation: An fMRI study of cognitive reappraisal in children, adolescents and young adults. Soc. Cogn. Affect. Neurosci..

[27] Schreiber L.R.N., Grant J.E., Odlaug B.L. (2012). Emotion regulation and impulsivity in young adults. J. Psychiatr. Res..

[28] Fino E., Melogno S., Iliceto P., D’Aliesio S., Pinto M.A., Candilera G., Sabatello U. (2014). Executive functions, impulsivity, and inhibitory control in adolescents: A structural equation model. Adv. Cogn. Psychol..

[29] Goldstein R.Z., Volkow N.D. (2011). Dysfunction of the prefrontal cortex in addiction: Neuroimaging findings and clinical implications. Neuroscience.

[30] Leyton M., Vezina P. (2014). Dopamine ups and downs in vulnerability to addictions: A neurodevelopmental model. Trends Pharm. Sci..

[31] Reifman A., Arnett J.J., Colwell M.J. (2007). Emerging adulthood: Theory, assessment, and application. J. Youth Dev..

[32] Steinberg L. (2007). Risk taking in adolescence: New perspectives from brain and behavioral science. Curr. Dir. Psychol. Sci..

[33] Aldao A., Nolen-Hoeksema S., Schweizer S. (2010). Emotion-regulation strategies across psychopathology: A meta-analytic review. Clin. Psychol. Rev..

[34] Buckner J.D., Keough M.E., Schmidt N.B. (2007). Problematic alcohol and cannabis use among young adults: The roles of depression and discomfort and distress tolerance. Addict. Behav..

[35] Gorka S.M., Ali B., Daughters S.B. (2012). The role of distress tolerance in the relationship between depressive symptoms and problematic alcohol use. Psychol. Addict. Behav..

[36] Zale E.L., Maisto S.A., Ditre J.W. (2015). Interrelations between pain and alcohol: An integrative review. Clin. Psychol. Rev..

[37] Kaiser A.J., Milich R., Lynam D.R., Charnigo R.J. (2012). Negative urgency, distress tolerance, and substance use among college students. Addict. Behav..

[38] Sher K.J., Grekin E.R., Gross J.J. (2012). Alcohol and affect regulation. Handbook of Emotion Regulation.

[39] Dingle G.A., da Costa Neves D., Alhadad S.S.J., Hides L. (2018). Individual and interpersonal emotion regulation among adults with substance use disorders and matched controls. Br. J. Clin. Psychol..

[40] Chambers R.A., Taylor J.R., Potenza M.N. (2003). Developmental neurocircuitry of motivation in adolescence: A critical period of addiction vulnerability. Am. J. Psychiatry.

[41] Brodbeck J., Matter M., Page J., Moggi F. (2007). Motives for cannabis use as a moderator variable of distress among young adults. Addict. Behav..

[42] Paulus D.J., Ditre J.W., Viana A.G., Bakhshaie J., Garza M., Valdivieso J., Ochoa-Perez M., Lemaire C., Zvolensky M.J. (2017). Pain and alcohol use among latinos in primary care: Examining rumination as an explanatory factor. Subst. Use Misuse.

[43] Price J.S., McQueeny T., Shollenbarger S., Browning E.L., Wieser J., Lisdahl K.M. (2015). Effects of marijuana use on prefrontal and parietal volumes and cognition in emerging adults. Psychopharmacology.

[44] Squeglia L.M., Jacobus J., Tapert S.F. (2014). The effect of alcohol use on human adolescent brain structures and systems. Handb. Clin. Neurol..

[45] Huh D., Mun E.Y., Larimer M.E., White H.R., Ray A.E., Rhew I.C., Kim S.Y., Jiao Y., Atkins D.C. (2015). Brief motivational interventions for college student drinking may not be as powerful as we think: An individual participant-level data meta-analysis. Alcohol. Clin. Exp. Res..

[46] Sinha R. (2008). Chronic Stress, drug use, and vulnerability to addiction. Ann. N. Y. Acad. Sci..

[47] Fishbein D., Miller S., Herman-Stahl M., Williams J., Lavery B., Markovitz L., Johnson M. (2016). Behavioral and psychophysiological effects of a yoga Intervention on high-risk adolescents: A randomized control trial. J. Child Fam. Stud..

[48] Aldwin C.M. (2007). Stress, Coping and Development.

[49] Russell B.S., Lincoln C.R., Starkweather A. (2018). Distress tolerance as a theoretical mechanism of action for the self-management of chronic conditions. J. Holist. Nurs..

[50] Russell B.S., Park C.L. (2018). The role of emotion regulation in chronic pain self-management. Top. Pain Manag..

[51] Gratz K.L., Weiss N.H., Tull M.T. (2015). Examining emotion regulation as an outcome, mechanism, or target of psychological treatments. Curr. Opin. Psychol..

[52] Miller A.L., Smith H.L., Hashim B.L., Kendall P.C. (2012). Dialectical Behavior Therapy with Multiproblem Adolescents.

[53] Ritschel L.A., Lim N.E., Stewart L.M. (2015). Transdiagnostic applications of DBT for adolescents and adults. Am. J. Psychother..

[54] Hsu S.H., Collins S.E., Marlatt G.A. (2013). Examining psychometric properties of distress tolerance and its moderations of mindfulness-based relapse prevention effects on alcohol and other drug use outcomes. Addict. Behav..

[55] Leyro T.M., Zvolensky M.J., Bernstein A. (2010). Distress tolerance and psychopathological symptoms and disorders: A review of the empirical literature among adults. Psychol. Bull..

[56] Kabat-Zinn J. (1994). Wherever You Go, There You Are: Mindfulness Meditation in Everyday Life.

[57] Bandura A. (1977). Self-efficacy: Toward a unifying theory of behavioral change. Psychol. Rev..

[58] Cooper D., Yap K., Batalha L. (2018). Mindfulness-based interventions and their effects on emotional clarity: A systematic review and meta-analysis. J. Affect. Dis..

[59] Davis J.M., Mills D.M., Stankevitz K.A., Manley A.R., Majeskie M.R., Smith S.S. (2013). Pilot randomized trial on mindfulness training for smokers in young adult binge drinkers. BMC Complement. Alten. Med..

[60] Himelstein S., Saul S., Garcia-Romeu A. (2015). Does mindfulness meditation increase effectiveness of substance abuse treatment with incarcerated youth? A pilot randomized controlled trial. Mindfulness.

[61] Price C.J., Wells E.A., Donovan D.M., Rue T. (2012). Mindful awareness in body-oriented therapy as an adjunct to women’s substance use disorder treatment: A pilot feasibility study. J. Subst. Abuse Treat..

[62] Sancho M., De Gracia M., Rodríguez R.C., Mallorquí-Bagué N., Sánchez-González J., Trujols J., Sánchez I., Jiménez-Murcia S., Menchón J.M. (2018). Mindfulness-based interventions for the treatment of substance and behavioral addictions: A systematic review. Front. Psychiatry.

[63] Vinci C., Peltier M.R., Shah S., Kinsaul J., Waldo K., McVay M.A., Copeland A.L. (2014). Effects of a brief mindfulness intervention on negative affect and urge to drink among college student drinkers. Behav. Res. Ther..

[64] De Dios M.A., Herman D.S., Britton W.B., Hagerty C.E., Anderson B.J., Stein M.D. (2012). Motivational and mindfulness intervention for young adult female marijuana users. J. Subst. Abuse Treat..

[65] Bornovalova M.A., Gratz K.L., Daughters S.B., Hunt E.D., Lejuez C.W. (2012). Initial RCT of a distress tolerance treatment for individuals with substance use disorders. Drug Alcohol Depend..

[66] Brown R.A., Lejuez C.W., Kahler C.W., Strong D.R., Zvolensky M.J. (2005). Distress tolerance and early smoking lapse. Clin. Psychol. Rev..

[67] Daughters S.B., Lejuez C.W., Bornovalova M.A., Kahler C.W., Strong D.R., Brown R.A. (2005). Distress tolerance as a predictor of early treatment dropout in a residential substance abuse treatment facility. J. Abnorm. Psychol..

[68] Daughters S.B., Lejuez C.W., Kahler C.W., Strong D.R., Brown R.A. (2005). Psychological distress tolerance and duration of most recent abstinence attempt among residential treatment-seeking substance abusers. Psychol. Addict. Behav..

[69] Hasan N.S., Babson K.A., Banducci A.N. (2015). The prospective effect of perceived and laboratory indices of distress tolerance on cannabis use following a self-guided quit attempt. Psychol. Addict. Behav..

[70] McHugh R.K., Weiss R.D., Cornelius M., Martel M.O., Jamison R.N., Edwards R.R. (2016). Distress intolerance and prescription opioid misuse among patients with chronic pain. J. Pain.

[71] O’Cleirigh C., Ironson G., Smits J.J. (2007). Does distress tolerance moderate the impact of major life events on psychosocial variables and behaviors important in the management of HIV?. Behav. Ther..

[72] Stein M.D., Herman D.S., Moitra E., Hecht J., Lopez R., Anderson B.J., Brown R.A. (2014). A preliminary randomized controlled trial of a distress tolerance treatment for opioid dependent persons initiative Buprenorphine. Drug Alcohol Depend..

[73] Gard T., Noggle J.J., Park C.L., Vago D.R., Wilson A. (2014). Potential self-regulatory mechanisms of yoga for psychological health. Front. Hum. Neurosci..

[74] Birdee G.S., Legedza A.T., Saper R.B., Bertisch S.M., Eisenberg D.M., Phillips R.S. (2008). Characteristics of yoga users: Results of a national survey. J. Gen. Intern. Med..

[75] Menezes C.B., Dalpiaz N.R., Kiesow L.G., Sperb W., Hertberg J., Oliveira A.A. (2015). Yoga and emotion regulation: A review of primary psychological outcomes and their physiological correlates. Psychol. Neurosci..

[76] Park C.L., Lee S.Y., Finkelstein-Fox L., Sanderson K., Plante T.G. (2018). Yoga to promote physical, mental and spiritual well-being: Self-regulation on and off the mat. Healing with Spiritual Practices: Proven Techniques for Disorders from Addictions and Anxiety to Cancer and Chronic Pain.

[77] Wheeler A., Wilkin L. (2007). A study of the impact of Yoga Âsana on perceived stress, heart rate, and breathing rate. Int. J. Yoga Ther..

[78] Eastman-Mueller H., Wilson T., Jung A.K., Kimura A., Tarrant J. (2013). iRest Yoga-Nidra on the college campus: Changes in stress, depression, worry, and mindfulness. Int. J. Yoga Ther..

[79] Goodman F.R., Kashdan T.B., Mallard T.T., Schumann M. (2014). A brief mindfulness and yoga intervention with an entire NCAA Division I athletic team: An initial investigation. Psychol. Conscious. Theory Res. Pract..

[80] Khanna S., Greeson J.M. (2013). A narrative review of yoga and mindfulness as complementary therapies for addiction. Complement. Ther. Med..

[81] Raina N., Chakraborty P.K., Basit M.A., Samarth S.N., Singh H. (2001). Evaluation of yoga therapy in alcohol dependence syndrome. Indian J. Psychiatry.

[82] Shaffer H.J., LaSalvia T.A., Stein J.P. (1997). Comparing Hatha yoga with dynamic group psychotherapy for enhancing methadone maintenance treatment: A randomized clinical trial. Altern. Ther. Health Med..

[83] Reddy S., Dick A.M., Gerber M.R., Mitchell K. (2014). The effect of a yoga intervention on alcohol and drug abuse risk in veteran and civilian women with posttraumatic stress disorder. J. Complement. Altern. Med..

[84] Falsafi N. (2016). A randomized controlled trial of mindfulness versus yoga: Effects of depression and/or anxiety in college students. J. Am. Psychiatr. Nurses Assoc..

[85] Yoga Journal & Yoga Alliance 2016 Yoga in America Study. http://www.yogajournal.com/yogainamericastudy/.

[86] Danhauer S.C., Addington E.L., Sohl S.J., Chaoul A., Cohen L. (2017). Review of yoga therapy during cancer treatment. Support Care Cancer.

[87] Macy R.J., Jones E., Graham L.M., Roach L. (2018). Yoga for trauma and related mental health problems: A meta-review with clinical and service recommendations. Trauma Violence Abuse.

[88] Cramer H., Ward L., Steel A., Lauche R., Dobos G., Zhang Y. (2016). Prevalence, patterns, and predictors of yoga use: Results of a US nationally representative survey. Am. J. Prev. Med..

[89] Tripathi M.N., Kumari S., Ganpat T.S. (2018). Psychophysiological effects of yoga on stress in college students. J. Educ. Health Promot..

[90] National Center for Educational Statistics The Condition of Education: College Enrollment Rates. https://nces.ed.gov/programs/coe/indicator_cpb.asp.

[91] Hopson L., Wodarski J.S., Tang N. (2015). The effectiveness of electronic approaches to substance abuse prevention for adolescents. E-Therapy for Substance Abuse and Co-Morbidity.

[92] Johnson C.C., Taylor A.G., Anderson J.G., Jones R.A., Whaley D.E. (2014). Feasibility and acceptability of an Internet-based, African dance-modified yoga program for African-American women with or at risk for metabolic syndrome. J. Yoga Phys. Ther..

